# Prevalence of Toothache and Associated Factors: A Population-Based Study in Southeast Iran

**Published:** 2013-08-01

**Authors:** Shahla Kakoei, Masoud Parirokh, Nouzar Nakhaee, Forogh Jamshidshirazi, Maryam Rad, Sina Kakooei

**Affiliations:** a Oral and Dental Diseases Research center, Kerman University of Medical Sciences, Kerman, Iran; b Neurosciences Research Center, Kerman University of Medical Sciences, Kerman, Iran

**Keywords:** Dental Health Services, Dental Hygiene, Education status, Socioeconomic status, Epidemiology, Iran, Pain, Prevalence, Toothache

## Abstract

**Introduction:**

This study was carried out to estimate toothache prevalence among adult residents in Kerman.

**Materials and Methods:**

This cross-sectional, population-based study was conducted among individuals aged over 18 years (*n*=1800). The relevant data on the prevalence of toothache and associated factors were collected by interviewing the individuals in their homes and filling out a questionnaire designed by the examiners. Prevalence of toothache and associated factors that patients recalled previous to their interview were analyzed by chi-square test and multivariate logistic regression analysis.

**Results:**

Nine hundred ninety-one individuals (55.1%) reported toothache during the 6 months before the interview. The participants who flossed daily, had regular dental visits, and had higher education showed a significantly lower prevalence of toothache (*P*<0.05), whereas regular tooth brushing and economic level of residency had no significant effect on the prevalence of toothache. Individuals between the ages of 26 and 45 [odds ratio (OR)=2.0], with a family size of more than 4 (OR=1.5), not using dental floss (OR=1.5), or having a mental or psychological illness (OR=1.5) were more likely to have a history of toothache.

**Conclusion:**

High prevalence of toothache (more than half) among residents of Kerman shows a serious and major public health problem. Toothache prevalence in middle aged adults, lower education, bigger family size, lower dental hygiene habit and/or those having mental or psychological illness were more common in the city of Kerman.

## Introduction

Toothache is the most common cause of oral pain [1]. Although fractured teeth and exposed dentin may produce dentin hypersensitivity and cause dental pain [2],untreated dental decay has been reported as the most important reason for toothache which can impact routine daily activities such as eating, studying, concentrating on delicate tasks, and so on [3-7].

Several investigations that studied the impact of dental and facial pain emphasized that tooth and mouth diseases directly influence the quality of life in a community [7, 8].

A wide range of toothache prevalence has been reported from 5% to 88% [9-11]. Dental pain has been confirmed as a public health problem [12]. A recent investigation of children and adolescents revealed that overall, about one-tenth of patients complaining of pain suffered from toothache [13].

Despite the increase in the number of dental schools and general dentists, as well as advanced educational programs during the past decade in Iran, the epidemiological profile of dental caries has not changed. It has been shown that a moderate-to-strong correlation exists between toothache prevalence and dental caries experience [14]. As toothache is the most common result of dental caries, we may conclude that most people who suffer from dental pain have had dental caries.

Data on the distribution and psychosocial effects of toothache should be considered when assessing the prevalence of dental pain among individuals in a community [9]. Studies have been conducted on community health and its trends to evaluate the effect of health care programs [15, 16].

Toothache has a deleterious impact on daily activities. In the United States alone, about 15 million working days are lost each year because of toothache [17].An investigation that evaluated the effect of oral health on people in the United Kingdom in 1998 reported that 51% of adult individuals were affected negatively by dental problems [18]. It can be assumed that because of the lack of previously published studies on the prevalence of toothache in Iran, little attention has been focused on this subject.

Based on our electronic search of Iranian (SID, Iranmedex) and PubMed electronic data banks using broad keywords up to 20 December 2012, the prevalence of toothache among the Iranian population was not reported. Therefore, we undertook to investigate the frequency and characteristics of toothache in a population-based study in the city of Kerman as the largest city in southeast of Iran.

## Material and Methods

A cross-sectional, population-based study was conducted in the city of Kerman which lies at the center of the largest province in southeast Iran. The study protocol was approved by the Ethics Committee of the Kerman University of Medical Sciences (No. KA 88/217). The survey utilized a 2-stage, random cluster sampling method. Samples include 1728 individuals based on the following parameters of toothache prevalence in developing countries: 10% [19], precision=2%, and design effect=2. At the end, 1850 questionnaires were prepared.

Kerman University of Medical Sciences is responsible for all health services in the city of Kerman, and it has health service centers in thirty three parts of the city. In this study, 10 health service sectors from health service center of Kerman University of Medical Sciences were randomly selected to include all economic levels of the population. The fieldwork was conducted by a team of 10 examiners who were trained, calibrated for the study and randomly checked by a professional supervisor and a dental student. 

Streets were selected at random, proportional to the number of street blocks. In each sector, 92 houses or apartments located on different streets were randomly selected. In each home, 2 individuals over 18 years old having at least 1 tooth during the prior 6 months were randomly interviewed. If nobody was at home, a second attempt was made 1 day later, if not, 1 of the neighbors was randomly chosen and interviewed. For households having only 1 individual older than 18, one of the neighbors were invited to participate in the study. Those with a mental disability who could not adequately communicate with the interviewer were excluded.

All participants were interviewed by a structured questionnaire that was developed according to previously conducted studies. [11, 20]. The content validity of the questionnaire was deemed appropriate by an expert panel and literature review. The variables included gender, age, educational status, area of residency, insurance coverage, smoking habits, family size, regular brushing and flossing habits, mouth wash use, frequency of routine dental visits, presence of systemic disease, history of toothache during the 6 months preceding the interview, pain-initiating factors, type of pain (sharp, pulsing, dull, continuous, night pain, diffuse, localized), and medication used for the pain relief and pain intensity (weak, intermediate, severe, very severe).

The data were analyzed using the chi-square test and the multivariate logistic regression analysis. P values less than 0.05 were considered significant.

## Results

One-thousand eight hundred participants of 1850 responded to the examiners (a response rate of 97.3%).

Approximately 513 (28.5%) were ≥25 years and 874 patients (48.6%) were aged between 26 to 45 years, and 20.9% of patients (377) were between 46 to 65 years. The rest were above 65 years old. The baseline characteristics are shown in Table 1. 

**Table 1.** Characteristics of the study subjects (n=1800)

Variable	Number	Percent
**Age**		
**≤25**	513	28.5
**26-45**	874	48.6
**46-65**	377	20.9
**>65**	25	1.4
**Missing**	11	0.6
**Gender**		
**Male**	701	38.94
**Female**	1077	59.84
**Missing**	22	1.22
**Number of family members**		
**<4**	1388	77.1
**≥4**	381	21.2
**Missing**	31	1.7
**Level of education of biological father**		
**Illiterate/primary**	327	18.2
**Secondary school**	416	23.1
**Diploma**	592	32.9
**Academic**	449	24.9
**Missing**	16	0.9
**Level of education of main person**		
**Illiterate/primary**	209	11.6
**Secondary school**	421	23.4
**Diploma**	626	34.8
**Academic**	503	27.9
**Missing**	41	2.3
**Insurance coverage **		
**Yes**	1458	81.0
**No**	335	18.6
**Smoking**		
**Yes**	167	9.3
**No**	1444	80.2
**Missing**	189	10.5

Of the 1800 interviewees, 55.1% reported toothache during the preceding 6 months. No significant prevalence of toothache was found based on gender, insurance coverage, economic level (categorized by previous studies), mouth wash usage, or brushing habits (*P*>0.05). However, there was lower toothache prevalence in individuals living in families with fewer members, higher level of participants education, dental flossing habits, regular dental visits, and having no history of systemic or mental disorders (Table 2) (*P*<0.05). Furthermore, there was significantly lower toothache prevalence in individuals above age 65 (*P*<0.0001).

**Table 2.** History of toothache during past six months according to selected variables

Variable	History of toothache during past six months		*P*-value
	Yes (%)	No (%)	
**Age**			<0.001
**≤25**	242 (47.4)	269 (52.6)	
**26-45**	523 (60.4)	343 (39.6)	
**46-65**	215 (57.8)	157 (42.2)	
**>65**	7 (33.3)	14 (66.7)	
**Gender**			0.684
**Male**	388(56.3)	301 (43.7)	
**Female**	592(55.3)	478 (44.7)	
**Family size**			0.003
**<6**	738 (53.8)	634 (46.2)	
**≥6**	236 (62.4)	142 (37.6)	
**Education of father **			0.001
**Illiterate/primary**	189 (58.7)	133 (41.3)	
**Secondary school**	257 (63)	151 (37)	
**Diploma**	315 (53.4)	257 (46.6)	
**Academic**	223 (50)	223 (50)	
**Education of respondent**			0.008
**Illiterate/primary**	123 (59.7)	83 (40.3)	
**Secondary school**	250 (60.7)	162 (39.3)	
**Diploma**	349 (56)	274 (44)	
**Academic**	250 (50.1)	249 (49.9)	
**Insurance coverage **			0.602
**Yes**	804 (55.3)	649 (44.7)	
**No**	185 (56.9)	140 (43.1)	
**Economic level**			0.457
**Low**	195 (54)	166 (46)	
**Intermediate**	494 (55)	404 (45)	
**High**	302 (57.9)	220 (42.1)	
**Smoking**			0.062
**Yes**	103 (63.2)	60 (36.8)	
**No**	796 (55.5)	637 (44.5)	
**Brushing and toothpaste usage**			0.150
**Yes**	923 (55.3)	747 (44.7)	
**No**	62 (62.5)	39 (37.5)	
**Dental floss usage**			<0.001
**Yes**	298 (48.7)	314 (51.3)	
**No**	682 (60)	455 (40)	
**Regular dental visits**			<0.001
**Yes**	577 (81.8)	128 (18.2)	
**No**	381 (40.2)	567 (56.8)	
**Mouthwash usage**			0.729
**Yes**	298 (56.3)	231 (43.7)	
**No**	683 (55.4)	549 (44.6)	
**Psychological or mental disease**			<0.001
**Yes**	206 (65.2)	110 (34.8)	
**No**	781 (53.9)	668 (46.1)	

The most prevalent type of pain intensity reported by respondents was ‘severe’ (34.4%) and the prevalence of sharp (29.5%) nd localized pain (29.9%) was more frequent compared with other types of pain perception (Table 3). 

The most frequently reported pain in terms of initiating factors was spontaneous pain (47.8%). Of all individuals who reported toothache, 74.5% visited a dentist when they were in pain. The most common medication used was analgesics (58.5%), antibiotics (18.9%), and local anesthetics (10.7%).

**Table 3.** Frequency of pain-related variables in patients with toothache

Variable	N	%
**Pain intensity**		
**Weak**	258	26
**Intermediate**	305	30.8
**Severe**	341	34.4
**Very severe**	79	8
**Missing**	8	0.8
**Initiating factor of pain**		
**Sensitivity to cold **	147	14.8
**Sensitivity to heat**	111	11.2
**Sensitivity to sweet material**	206	20.8
**Sensitivity to pressure and chewing**	181	18.3
**Spontaneous**	474	47.8
**Type of pain**		
**Sharpen**	292	29.5
**Pulsing**	95	9.6
**Dull**	140	14.1
**Continuous**	133	13.4
**Night pain**	128	12.9
**Generalized**	70	7.1
**Localized**	296	29.9
**Drug consumption**	777	78.4
**Analgesic**	580	58.5
**Antibiotics**	187	18.9
**Localized drug**	31	3.1
**Remedies**	70	7.1
**Anesthesia drug**	106	10.7
**Self- prescription**	567	57.2
**Dentist prescription**	218	22
**Seen dentist during pain**	738	74.5
**Treatment plan of dentist **		
**Extraction of teeth**	314	31.7
**Restoration of teeth**	352	35.5
**Both of them**	52	5.2
**Others**	273	27.5

Among those reporting toothache during the 6 months preceding the interview, 57.2% used medication without a dentist’s prescription and 22% did so after visiting a dentist.

Using multivariate logistic regression, only 4 variables, including age group [odds ratio (OR)=2], family size (OR=1.5), dental floss usage (OR=1.5), and presence of systemic or mental disease (OR=1.5), showed significant association with the history of toothache during the prior 6 months (Table 4). 

**Table 4.** Adjusted odds ratios for history of toothache among 1800 residents of Kerman

Variable	OR	95% CI	*P*-value
**Age**			
**≤25**	Ref.	--	--
**26-45**	2.0	1.5-2.6	<0.001
**46-65**	1.5	1.1-2.1	0.02
**>65**	0.60	0.2-1.9	0.39
**Family size**			
**<4**	Ref.	--	--
**≥4**	1.5	1.2-2.0	0.002
**Education of father **			
**Illiterate/primary**	Ref.	--	--
**Secondary school**	1.2	0.8-1.7	0.34
**Diploma**	0.8	0.6-1.2	0.26
**Academic**	0.8	0.6-1.3	0.41
**Education of respondent**			
**Illiterate/primary**	Ref.	--	--
**Secondary school**	1.2	0.8-1.9	0.37
**Diploma**	1.3	0.8-2.0	0.30
**Academic**	1.2	0.8-2.0	0.36
**Smoking**			
**No**	Ref.	--	--
**Yes**	1.1	0.7-1.5	0.77
**Brushing and toothpaste usage**			
**Yes**	Ref.	--	--
**No**	0.95	0.6-1.5	0.84
**Dental floss usage**			
**Yes**	Ref.	--	--
**No**	1.5	1.2-1.8	0.001
**Psychological or mental disease**			
**No**	Ref.	--	--
**Yes**	1.5	1.1-2.0	0.01

## Discussion

This study examined the frequency and characteristics of toothache among residents of Kerman, Iran. In the study, 1800 individuals were interviewed, and a staggering 55.1% reported toothache during the preceding 6 months.

Before interpreting the results, we should bear in mind the limitations of a cross-sectional study: the possibility of recall bias should not be overlooked. Any association between the analyzed variables might be non-causal. Table 4 shows that we have used multivariate logistic regression which is included amongst advanced regression models. 

Previous studies have investigated either orofacial pain or toothache individually [20-37].This investigation focused only on toothache. The high percentage of toothache prevalence (55.1%) herein supports the importance of focusing on toothache since orofacial pain is considered a wider subject. Thus, in addition to toothache, other disorders such as temporomandibular joint disorders, migraine headache, neuralgia, oral ulcers, oral sores, burning mouth, pain in mastication of muscular origin, and muscular pain in the head and neck should also be considered independently [21-25]. However, these disorders may mimic toothache and therefore the actual percentage of pain with dental origin might be less than what was reported by the respondents. Several factors may influence a dental pain report, such as socioeconomic factors, gender, age, and level of education [4, 20, 37]. In this study, people over 65 showed the lowest prevalence of toothache, whereas individuals between 25 and 65 showed the highest prevalence. No unique pattern of pain has been reported among different ages in previous investigations of toothache. In one study, 11.7% of children and adolescents experienced tooth pain during the preceding 6 months [13]. In contrast; another investigation reported that 63% suffered pain over a lifetime [31]. The major reason for lower prevalence of toothache in over 65’s may be that they have less teeth remaining. Previous investigations have confirmed a higher prevalence of toothache in younger individuals [20, 34].

The results of the present study revealed that there is no difference in gender among individuals who experienced pain during the previous 6 months. There are debatable results regarding the gender and prevalence of toothache. Some previous investigations in many countries reported a higher number of females having experienced toothache during a certain period of time [20, 28]. In contrast, Pau *et al.* in a review of articles published during 1996-2001, detected no significant relation between gender and toothache[9].

Educational and social level may play an important role in toothache prevalence [10, 38], because better-educated people seem to take better care of their teeth, and the health of their family is more important to them than those with little or no education. The results of the present study showed that individuals with an academic educational level had a significantly lower experience of toothache compared with those who were illiterate or had only a primary education (*P*<0.008). This finding is in agreement with a similar study that has performed on the Australian indigenous communities on the prevalence of toothache [10].

Smoking has been linked to dental and oral health status [39]. Some investigators have reported a significant relation between smoking behavior [11, 30, 31, 33]. In the present study, there was a measurable, but not significant, relation (*P*=0.062) between smoking and toothache during the 6 months before the interview.

Similarly, we found that bigger family size had a significant effect on toothache prevalence (P<0.002, O=1.5); the bigger the family the more toothache experienced. Other investigators, however, have not addressed this issue.

Previous investigations have emphasized that welfare has a significant effect on toothache experience in a population [4, 9, 32, 34-37]. However, in our investigation, because the authors were not confident enough to get precise information regarding individuals’ income, a different strategy was used by sending the interviewers to all economic levels of Kerman, with the finding of no significant difference between different income levels with respect to toothache (*P*>0.05). Other studies have shown that an unhealthy lifestyle may directly influence dental health [40, 41]. Wealthy sections of Kerman contain numerous fast-food facilities that serve soft drinks with each meal. Soft-drinks in Iran have shown that most of these beverages have a high acidic pH [42, 43]. The roles of acidic pH and high carbohydrate content of soft drinks on increasing the prevalence of tooth decay is well established [40, 41].

One of the possible causes of conflicting results in pain prevalence studies is asking the history of pain experience before the interview. Several studies have investigated lifetime prevalence [31], while many others have focused on pain prevalence during the prior 3 to 12 months before the interview [11, 20, 28]. We investigated pain experience during the preceding 6 months to the interview, which was considered to be the highest pain prevalence among studies covering the same preliminary period [11, 20]. It has been stated that the varying results found in toothache prevalence investigations may be due to methodological differences such as sample size and gender as well as the uniqueness of each community/culture [20].

Likewise, the prevalence of toothache might be influenced by referred pain of other than dental origin, which is a common finding in patients having no real dental problem seeking dental treatment [44]. Differential diagnosis should be performed by a skilled, well-trained dental practitioner doing comprehensive radiographic and clinical examinations. However, through the quality of pain questioned in the present study, we have shown that respondents having sharp pain that is spontaneous, localized, and pulsing were much more likely to have toothache than respondents who reported continuous burning pain. It was not possible to examine all respondents extensively; in any case, most previously performed investigations used the same self-reporting method for assessing the prevalence of toothache in their society [11, 20, 28, 30, 31, 34-37].

Most of the respondents who experienced pain (74.5%) had to visit a dentist for either advice or treatment (Table 3). Without clinically examining our participants, it was not possible to determine the etiology of their pain. 

In this study, 206 out of 1800 (11.44%) individuals had psychological or mental disorders. There was significantly more toothache experience in these patients than in healthy individuals (*P*<0.01, OR=1.5). Our findings corroborated those of previous investigations [3, 45].

The improvement in life quality is a goal of all health care professionals, policy makers, and health care authorities. Because most oral diseases and their consequences interfere with daily life performance, it seems reasonable to obtain adequate data on both the prevalence of oral diseases and their impact on the daily activities of all individuals in a society [4]. Investigating the prevalence of toothache can give policymakers and health authorities the required data about a community and its health care needs.

## Conclusion

In conclusion, the results of the present study showed that more than half of the Kerman residents had a history of pain during the preceding 6 months. Age group, family size, dental floss usage, and systemic or mental disease were significantly correlated with pain experience.
